# Toward consensus: using the Delphi method to form an international expert consensus statement on ultra-processed food addiction

**DOI:** 10.3389/fpsyt.2025.1542905

**Published:** 2025-05-01

**Authors:** Jen Unwin, Heidi Giaever, Nicole Avena, Clarissa Kennedy, Molly Painschab, Erica M. LaFata

**Affiliations:** ^1^ The Collaborative Health Community Foundation, Oxford, United Kingdom; ^2^ Icahn School of Medicine at Mount Sinai, New York, NY, United States; ^3^ Princeton University, Princeton, NJ, United States; ^4^ Sweet Sobriety, Belgrade, MT, United States; ^5^ Center for Weight, Eating, and Lifestyle Science, Drexel University, Philadelphia, PA, United States

**Keywords:** ultra-processed food, addiction, substance use disorder, consensus, Delphi method

## Abstract

The recognition of an addictive disorder relating to the harmful use of certain foods is being called for by clinicians and researchers, which evidence supports as being distinct from eating disorders (EDs) and obesity. Critics cite a lack of consensus on the validity of associating the term addiction with food, claiming that characteristics of addiction ‘are not observed in the context of eating behaviors’ as a reason to dispute its consideration as a novel diagnosis. It was decided to consult international scientific and clinical experts to review whether it would be possible to reach a consensus around this subject. The 12-month project, using a Delphi method, involved 40 clinicians, researchers and academics, from 10 countries and a team of four facilitators. Consensus was achieved between 37 out of the 40 participants. The discussions during the process demonstrated that it was not only possible to achieve several areas of agreement related to the clinical observation of addiction-like symptoms related to certain foods but also, that existing extensive scientific research findings confirm the biochemical, neurological and behavioral aspects of a substance-use disorder relating specifically to ultra-processed foods, exhibiting strong similarities with other acknowledged substance-use disorders. The consensus may provide a platform for future attempts for formal recognition of ultra-processed food addiction as a diagnosis. Areas for future research are discussed.

## Graphical Abstract

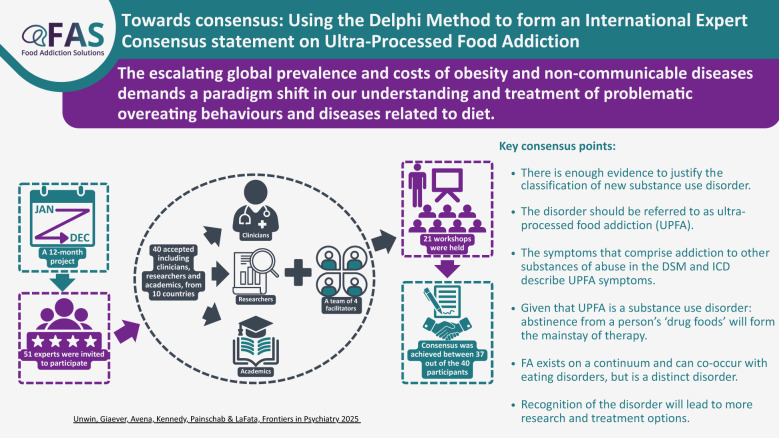

## Introduction

The escalating global prevalence and costs of obesity and non-communicable diseases, such as type 2 diabetes, cancer, dementia, poor mental health and fatty liver disease, demands a paradigm shift in our understanding and treatment of problematic overeating behaviors and diseases related to diet (1). An increasing awareness of the correlation between these diseases and the growing global consumption of ultra-processed foods (UPFs), and emerging evidence of causation, is leading to calls to action at many levels (2). The urgency for action is prompted by the growing recognition that UPFs, through their biochemical impact on the human brain, appear to cause overeating and induce addiction-like behaviors in susceptible individuals (for a review of the evidence on the addictive potentials of UPFs, see 3). Research and clinical experience have increasingly shown that these behaviors mirror the diagnostic indicators of substance-use disorders (e.g., use despite negative consequences, withdrawal) and are thought to be driven by the overstimulation and sensitization of the brain’s reward pathways by UPFs, akin to other known addictive substances (4).

In line with this growing evidence base, in March 2021, several members of this team made an initial submission to the World Health Organisation (WHO) to accept ‘the harmful use of foods’ as a mental disorder, like other substance use disorders, but related to certain types of foods as ‘the substance’. It was rejected by the Medical and Scientific Advisory Committee (MSAC) that advises the WHO on scientific updates to the International Classification of Disease, ICD-11. One of their concerns was the contentious nature of listing ‘food addiction’ as a disorder, fearing that referencing foods as being addictive could introduce “confusion and inappropriate use in clinical practice and public policies.” In response to this feedback, an international consensus building workshop program was undertaken from May 2023 to April 2024, involving clinicians, researchers, and academics representing 10 countries and all of whom had expertise with the construct of food addiction. Our workshops aimed to harness the collective insights of the experts, their scientific research and clinical experience, and to review what could be agreed about the subject of ‘addiction-like symptoms related to food.’

Demonstrating consensus among interdisciplinary experts was undertaken to advance three key domains related to the acceptance of addictive-like eating as a novel clinical presentation. First, achieving consensus around the theoretical and phenotypic features of ‘food addiction’ would increase scientific credibility of the construct. For instance, multiple variations of a similar term have been used to refer to the types of foods most implicated in ‘food addiction’ (e.g., ultra-processed foods, highly processed foods, sugar). While these terms largely refer to the same foods, the lack of consistent terminology has been consistently pointed to as evidence that ‘food addiction’ is a controversial topic without a clear definition. Thus, clarification of the terminology would likely increase scientific credibility and reduce controversy around this construct.

Second, achieving consensus in the conceptualization of ‘food addiction’ could have critical clinical implications, such as the development of standardized diagnostic criteria and intervention protocols to support reliable case conceptualization and evidence-based treatment planning. Importantly, if ‘food addiction’ could be recognized as a clinical presentation, affected individuals may feel validated by the recognition of their experiences and may have access to insurance-billable treatments for the first time.

Third, establishing consensus may advance policy initiatives and improve public understanding. For instance, agreement on which foods are associated with addictive-like eating is a mandatory precursor to the development of addiction-informed public policies, such as restricting marketing of these foods to children. Continuing with this example, specifying the foods most implicated in addictive-like eating could result in a narrower term than the misnomer ‘food addiction,’ which could improve public understanding that individuals uniquely develop addictive-like responses to foods not required for survival. Broadly, a unified stance simplifies messaging to both policymakers and the public, making it easier to convey the urgency and rationale for recognizing ‘food addiction’ as a clinical presentation and implementing policy initiatives to protect the public from the addictive potentials of highly reinforcing foods. Notably, agreeing upon the defined scope of a public health concern is a foundational step to motivate global health authorities, like the WHO, to take coordinated and strategic mitigating action.

The multifaceted implications that may follow from achieving consensus on the conceptualization of ‘food addiction’ motivated experts to participate in these workshops. This manuscript describes the process by which consensus was facilitated and details several domains for which consensus was achieved.

## Method

### Overview

The workshops began by determining what could already be agreed among the experts, which was largely reflective of the sound evidence for the biochemical, psychological, and social aspects of ‘food addiction’, as well as the recognition of which foods may be implicated in addictive-like eating. We hypothesized that there is sufficient scientific research and clinical observations to inform discussions among international experts on this topic that would result in a consensus definition of the condition. To develop this consensus definition of food addiction, we utilized the Delphi method (also known as estimate-talk-estimate). The Delphi method is a group facilitation technique designed to solicit individuals’ expert opinions and transform these perspectives into a group consensus (5–7). The method has been used previously to establish protocols for the use of a ketogenic diet in epilepsy (8).

The Delphi method relies on experts who are knowledgeable about a certain topic (in our case, ‘food addiction’ or ‘addiction-like’ symptoms related to ‘food’) and who are willing to work with other experts to review whether agreement (consensus) can be reached for a larger purpose. In our case, the larger purpose of a consensus definition of ‘food addiction’ is to support the recognition of this presentation as a novel diagnosis, which is a key step to facilitating subsequent treatment program development, implementation and evaluation of treatments, future research initiatives, and primary prevention.

### Participants and facilitators

The Delphi method participants (i.e., the experts) were clinicians, academics, independent researchers, and/or investigative journalists with extensive demonstrated experience (e.g., having published peer-reviewed papers, conference presentations, books, white papers, and/or expert opinion articles) on the topic of food addiction. Participants from any country were considered; however, given that most of the research on the intersection of ‘food’ and ‘addiction’ has been conducted in Europe and North America, *a priori* we were aware that participants were more likely to be from these regions. In the interest of reducing industry bias, we did not include experts working in the food or pharmaceutical industries.

Participants were identified by the Delphi facilitators and contacted initially by email. A convenience snowball sampling technique was used to extend the number of participants; this snowballing technique was based on the networks of experts the facilitators contacted. Given that the area of ‘food addiction’ research is relatively small, the facilitators did not anticipate inviting more than 50 participants. In total, 51 experts were approached and invited to participate, of which 40 accepted. The goal was to have a minimum of 30 participants consistent with de Villiers et al. (7) who suggest that a panel of more than 30 participants may not necessarily improve the quality of the Delphi result.

The Delphi facilitators were four food addiction professionals who have each been actively working and researching in the field for more than five years. The facilitators worked in pairs to facilitate online workshops and manage the Delphi process.

### Workshops

Participants who agreed to be involved in the consensus process, were invited to workshops to share their academic, research, and/or clinical experience. Each workshop involved 3–4 experts, and a total of 21 workshops were held for 11 groups. Participants were guided by a set of five questions sent to them ahead of the workshops (see **Box 1**) and were asked to answer these questions based on their expert opinion and present their responses in the workshop.

Box 1 **Questions used to guide experts in the consensus development.**

1. What should the disorder be called?
2. Can we agree on a definition?
3. What is the evidence (or lack thereof) in the research on the disorder:
  -as distinct from eating disorders (ED)
  -on treatment?
4. What is the evidence of similarities between Food Addiction (FA) and other dopamine driving, survival related behavior disorders like ‘sex addiction’, or other substance use abuse disorders?
5. Can the disorder be placed in one of the existing categories in ICD-11?

After participants answered the questions in the workshops and fellow participants were able to ask clarification questions, the facilitators collected the answers, presentations, and any references provided, and subsequently summarized, in a report, the answers for which consensus within the group was apparent. The experts were then asked to review the summary report and either agree or disagree with the consensus proposed in a second workshop, which resulted in the report being modified accordingly by the facilitators.

The experts were encouraged to discuss and share opinions and perspectives among themselves and were invited to finalize the consensus points within their workshop groups. As new groups of experts joined the program, the role of the facilitators was to host the workshops and then expand and modify the consensus as it appeared to develop.

### Final consensus development

After the final workshop concluded, a master consensus was compiled by the facilitators and shared with all 40 participating experts. There followed a process of discussions with individual experts to review outstanding concerns and questions until a master consensus was agreed.

## Results

Following the development of the master consensus proposed by the facilitators, a total of 37 out of the 40 participants were able to agree the summary. Of the three participants who could not agree the consensus, two considered the disorder to be primarily behavioral rather than a substance use disorder, and one could not agree the term Ultra-Processed Food Addiction.

The full agreed consensus statement and list of consensus-building workshop participants and facilitators are shown in the **Supplementary Materials A, B**. A summary of the final agreed consensus is shown in **Box 2**.

Box 2 **Consensus summary.**

1. Name
  The disorder should be called ultra-processed food addiction (UPFA).
  - There was much debate on this issue. Some participants favored food addiction, processed food addiction or sugar addiction, for example.
  - It was agreed that most research supports the term ultra-processed food addiction, given that ultra-processed foods have been most strongly associated with the diagnostic features of substance-use disorders.
2. Definition
*Ultra Processed Food Addiction (UPFA) is a chronic disease involving complex interactions among brain circuits, genetics, the environment and an individual’s life experiences. People with UPFA use ultra-processed foods in a way similar to drugs of abuse, obsess about ultra-processed foods, and/or engage in eating behaviors with ultra-processed foods that become compulsive and often continue despite harmful medical and biopsychosocial consequences.*
  The definition is a modified version of the ASAM definition of addiction (2019) to parallel theoretical and phenotypic features of addictive disorders.
3. Research
  The following was agreed regarding UPFA research to date.
  - There is sufficient evidence that people use ultra-processed foods in an addictive way (UPFA).
  - UPFA can occur with or without eating disorders (ED).
  - UPFA can also be comorbid with several disorders including T2D, CVD, Obesity, mental health disorders, chronic pain, and others.
  - Further research is needed on assessment protocols, treatment outcomes, phenomenology, risk factors and prevention.
4. Similarities with substance-use and addictive disorders
  - UPFA is a substance-use disorder, meaning it involves compulsive consumption of addictive ultra-processed foods and engagement in the behavioral criteria for diagnosing substance-use disorders (e.g., use despite negative consequences) related to consumption of these foods.
  - Comparators with known addictive substances include nicotine, caffeine, and alcohol.
  - Individuals abstaining from disordered use of UPFs can experience withdrawal symptoms (anxiety, irritability, insomnia, dysphoria, and craving).
  - Animal studies, human brain imaging studies, psychometric research (using YFAS, The Highly Processed Food Withdrawal Scale; PROWS), and large-scale epidemiological studies of UPFA show similar patterns with other addictive disorders.
  - UPFA meets the four criteria for a public health problem requiring societal intervention. Ubiquity, toxicity, abuse, negative impact on society (US criteria).
5. Can the disorder be placed in a current ICD 11 category
  - Broad agreement that UPFA requires a new subcategory within the substance use parent category.
  - UPFA symptoms are not fully accounted for in the eating disorder or obesity categories.

## Discussion

The collective understanding and substantial majority agreement demonstrated in this consensus process, underscores the necessity for further action to classify UPF addiction as a novel psychiatric disorder. Such a classification could offer critical opportunities in the research and clinical realms. Firstly, it could facilitate government investments into further research on the development of addiction-informed treatments for individuals experiencing the addictive-like intake of UPFs. Secondly, it would enable clinicians to formally diagnose clinically evidenced addiction-like symptoms related to UPFs and offer some of the emerging and successful biopsychosocial treatment options for this disorder. Thirdly, it could provide an informed and compelling platform from which preventative work could be launched, to reduce the risks of future generations being impacted by the negative health influences of UPFs and the consequential costs to society. Finally, it could lead to government policy decisions being made that would compel relevant parts of the food industry to take responsibility in much the same way as happened with the tobacco industry.

Consensus participants were able to agree on the existence of a unique disorder of UPFA, differentiated from but often co-morbid with EDs and obesity. There was agreement that the condition resembled other substance-use disorders. Further research is needed particularly regarding effective treatment protocols.

The agreed consensus provides the basis for a reply to the ICD-11 committee within the WHO, to request recognition of UPF addiction as a disorder. Furthermore, reviewing, prioritizing and raising funding for the research that the consensus workshops have concluded needs to be done and the development of successful treatment programs would follow from such a recognition.

Consensus participants agreed on a number of areas where future research efforts should be targeted. Firstly, further evidence-based protocols and tools are needed for the assessment of UPFA. One such semi structured clinical interview is now published (9). Such a protocol must include accounting for EDs and distinguishing between true positives and false positives in UPFA, identified when using screening tools. Research is also needed on UPFA treatment outcomes (including accounting for EDs), looking at different therapeutic modalities (including medication) that target UPFA as a biopsychosocial disorder. Further research is needed to identify the susceptibility to UPFA based on the following risk factors: genetics, epigenetics, environmental impact (ubiquity, marketing, social influence), effect of early and chronic exposure, relevant psychological factors, and the effects of legislation. This could lead to research on preventative interventions with children.

There are dissenting voices in both research and clinical practice who refute the idea of UPFA (e.g., 10 is commonly cited) and some who are calling for more research to establish such a condition. Notably, food industries would not be in favor of recognizing the addictive nature of UPFs (11). There is evidence that industry conflicts of interest are a significant issue in the literature. For example, Westwater et al. (10) was part of a supplement sponsored by Rippe Health. Dr. Rippe’s significant ties to industry sponsorship were exposed by the New York Times in 2104 (12).

The methodology of this consensus process has certain weaknesses. The use of the ‘snowball’ technique to recruit participants could have resulted in a lack divergent opinions. There have also been several criticisms of the Delphi method such as the lack of anonymity of the experts, which prevents a more dynamic and in-depth debate, the excessively subjective nature of the method, the lack of transparency, and its difficult reproducibility (13).

Furthermore, questions remain as to the possible harms of a new substance use diagnosis in terms of the excessive medicalization of behaviors and a certain amount of individual responsibility for these behaviors. Two of the participants were psychiatrists and were able to contribute to this aspect of the discussion.

The consensus process results demonstrate agreement among clinicians and researchers without ties to the food industry that it is possible to form broad agreement about the existence of a distinct condition of UPF addiction. All 40 participants agreed that the condition can be differentiated from other eating disorders and urgently requires the development of effective biopsychosocial assessments and interventions. Continuing to ignore the impact of the addictive properties of foods and the susceptibility of certain individuals, in certain circumstances, to getting ‘hooked’ on these foods, will risk the worsening of obesity, problematic patterns of overeating behavior, and chronic metabolic disease, affecting increasingly younger populations globally. The authors’ hope is that growing consensus will pave the way for official recognition of addiction-like symptoms related to certain foods as a substance use disorder, with an emphasis on UPFs as the ‘substance’. Such recognition will lead to the development of preventative strategies and effective treatment protocols that address the root causes of this condition rather than merely its symptoms.

## Data Availability

The original contributions presented in the study are included in the article/**Supplementary Material**. Further inquiries can be directed to the corresponding author.
