# Supplementary material for: Toward consensus: using the Delphi method to form an international expert consensus statement on ultra-processed food addiction
Source: Front Psychiatry. 2025 May 1;16:1542905. doi: 10.3389/fpsyt.2025.1542905 (PMC12078235; doi:10.3389/fpsyt.2025.1542905)
Supplement: Supplementary file 1 [file DataSheet1.zip › Supplementary Material B. Consensus Participants and Facilitators.docx]

**Supplementary Materials B**

CONSENSUS PARTICIPANTS

Erica LaFata, PhD

Dr. David Wiss, PhD, MS, RDN

Dr. Kim Dennis, MD, CEDS

Dr. Vera Tarman, MD, MSc, FCFP, ABAM

Dr. Timothy Brewerton, MD, DLFAPA, FAED, DFAACAP, CEDS-S

Dr. Claire Wilcox, MD

Connie Stapleton, PhD

Michael Moss

Dr. Eric Westman, MD, MHS

Dr. Robert Pretlow, MD, MSEE, FAAP

Dr. Georgia Ede, MD

Vincent Santiago, PhD

Dr. Robert Lustig, MD. MSL

Dr. Anna Lembke, MD

Dr. Tro Kalayjian, MD, DO

Dr. Adrian Soto-Mota, MD, PhD, FACP

Dr. Stephen Tate, MD

Dr. Ignacio Cuaranta, MD

Susan Peirce Thompson, PhD

Dorothea Portius, PhD

Dr. Evelyne Bourdua-Roy, MD

Stephan Guyenet, PhD

Dr. Tracy Burrows, PhD

Dr. Agnes Ayton, MD, FRCPsych, MMedSc, MSc

Fernando Fernandez-Aranda, PhD, FAED

Dr. Paul Earley, MD, DFASAM

Dr. David Cavan, MD, FRCP

Dr. Octavian Vasiliu, PhD, PsyD, MA, MSc

Amanda Leith, FAC

Theresa Wright, MS, RDN, LDN

Dr. Christina Horsager Pedersen, MD

Dr. Shebani Sethi, MD, ABOM

Dr. Nicole Avena, PhD

Anna Fruehling, CHPC, PHC

Dr. Irit Hochberg, MD

Dr. Mariela Glandt, MD

Dr. Joan Ifland, PhD

Bitten Jonsson, RN

Marty Lerner, PhD

Esther Helga Gudmundsdottir, MSc

FACILITATORS

Heidi Giaever, BSc

Dr. Jen Unwin, PsyD, FBPSs

Clarissa Kennedy, RSW

Molly Painschab, LCPC, LAC
