# Supplementary material for: Toward consensus: using the Delphi method to form an international expert consensus statement on ultra-processed food addiction
Source: Front Psychiatry. 2025 May 1;16:1542905. doi: 10.3389/fpsyt.2025.1542905 (PMC12078235; doi:10.3389/fpsyt.2025.1542905)
Supplement: Supplementary file 1 [file DataSheet1.zip › Supplementary Material A. Food Addiction Consensus Statement May 2024.docx]

**Supplementary Materials A**

**FA Consensus Workshops - Consensus Statement – May 2024**

(*) Note: Definitions at the end of the Consensus Statement

**Agreement on the following questions by 37 of 40 workshop participants:**

***Question 1****: The name of the disease of addiction symptoms related to Food (FA):*

- Ultra-Processed Food (UPF) Addiction
- Disorders due to use of UPF (ICD-11 terminology)

***Question 2****: How should it be defined?*

*(Please refer to* ***Definitions*** *section at the end of this document)*

- Use current ASAM definition with below edit (*) and SUD 11 criteria in DSM5 and Disorders due to Substance Use in ICD-11. More detailed review of ICD-11 to follow in subsequent workshops.
- Use Yale Food Addiction Scale (YFAS) 2.0 with the caveat that recent severe caloric restriction can cause false positives.
- Proposed modifications to ASAM Definition of Substance Use Disorders (SUDs) as it relates to Food:

*(*)Ultra Processed Food Addiction (UPFA) is a chronic disease involving complex interactions among brain circuits, genetics, the environment and an individual’s life experiences. People with UPFA use food in a way similar to drugs of abuse, obsess about food, and/or engage in eating behaviours that become compulsive and often continue despite harmful medical and biopsychosocial consequences.*

***Question 3****: Evidence and Research:*

*Existing Evidence and Research*

There is sufficient evidence that people use foods in an addictive way (UPFA).

- UPFA can occur with or without eating disorders (ED).
- UPFA can also be comorbid with several disorders including T2D, CVD, Obesity, mental health disorders, chronic pain, and others.
- Research demonstrates parallels and connections with other substances and SUD; including caffeine, alcohol and nicotine. As one example, new evidence suggests that GLP-1s reduce cravings of both alcohol and food, thereby corroborating a common pathway for both disorders related to the reward system.

*Research Needed*

- Symptom severity varies in both UPFA & ED and further research is necessary into biological and psychological factors and assessment protocols.
- Evidence based protocols are needed for assessment of UPFA. A clinical interview schedule is needed. This must include accounting for ED and distinguishing between true positives and false positives in UPFA, identified when using screening tools.
- Research is also needed on UPFA treatment outcomes (including accounting for ED), looking at different therapeutic modalities (including medication) that target UPFA as a biopsychosocial disorder.
- Clinical research is also needed on the phenomenology of UPFA (including accounting for ED) to study the development of addiction symptoms across the lifespan, including whether dietary restraint, body image / dissatisfaction, and cue reactivity drive compulsion, tolerance, withdrawal, neglect and progression.
- Research is needed on preventative interventions with children.
- Neurobiological and psychological research is needed as to the effect of consumption to understand the more detailed mechanistic aspects of the addictive agents in UPFA. Sugar and caffeine, and combinations of artificial sweeteners and sugars, are examples of rewarding substances that can increase the motivation to eat specific foods, potentially increasing the risk of addiction.
- Research is needed to identify the susceptibility to UPFA based on the following risk factors: genetics, epigenetics, environmental impact (ubiquity, marketing, social influence), effect of early and chronic exposure, relevant psychological factors, and the effects of legislation.
- Need for further development of scale/assessment tools.

***Question 4****. Evidence of similarities with addictive behaviour or other Substance Use Disorders (SUD)*

- UPFA is both a substance use and behavioural addiction, meaning it involves compulsive consumption of addictive foods and engagement in addictive behaviours related to consumption.
- Comparators with known addictive substances include nicotine, caffeine, and alcohol.
- Individuals abstaining from disordered use of UPFs can experience withdrawal symptoms (anxiety, irritability, insomnia, dysphoria, and craving).
- Animal studies, human brain imaging studies, psychometric research (using YFAS, The Highly Processed Food Withdrawal Scale; PROWS), and large-scale epidemiological studies of UPFA show similar patterns with other addictive disorders.
- There are developed, validated scales and assessment tools (YFAS, PROWS).
- UPFA meets the four criteria as a public health problem requiring societal intervention. Ubiquity, toxicity, abuse, negative impact on society (US criteria).

***Question 5****. ICD-11 Category:* (More detailed review of ICD-11 to follow in subsequent workshops)

- Broad agreement to use SUD parent
- A separate category in 6C is proposed, or to use 6C4 (Substance Use) and/or 6C5 (Addictive Behaviours).
- Another suggestion is category 6C4E Disorders due to use of other specified psychoactive substances, including medications. Subcategories w/ wo ED.

Note: There is currently no 6C6 category. 6C7 is Impulse Control Disorders.

- To be reviewed in the next phase of the consensus process.

***Additional Observations and Conclusions from Consensus Workshops***

The signatories to this document support the clinical evidence that suggests that UPFA is a treatable, chronic medical disease involving complex interactions among brain circuits, genetics, the environment, and an individual’s life experiences. Furthermore, UPFA represents a significant challenge to public health by its links to Obesity, T2D, mental health disorders and other conditions. UPFA is uniquely difficult to treat due to early exposure, ubiquitous access, social encouragement, cultural norms, and multi-billion-dollar marketing campaigns. Like treatment of people with other SUDs, abstinence from UPF is increasingly seen as the best course of action for most people with UPFA. Others may be able to moderate certain foods. Clinical experience suggests that people with UPFA may also need to abstain from other, non-UPF, triggering foods. Both abstinence from the ‘substance’ but also abstinence from the behaviour (e.g. volume, overeating, snacking or restricting) should be considered and related to the specificity of the individual’s phenotype and wishes. Clinical experience also indicates that a real whole food dietary treatment for UPFA, ensuring optimum nutrition content, is likely also to benefit patients with ED, including AN. UPFA may explain some of the 30% of people in ED treatment that never get better. An all-foods-fit approach for a person with UPFA is likely to be an ineffective treatment.

Further work is needed to define UPF as there are some limitations to the current NOVA system. This work was beyond the scope of the current project. See also ***Definitions*** below.

***Definitions:***

**American Society of Addiction Medicine (ASAM) definition of addiction** (2019)

Source: <https://www.asam.org/>

Addiction is a treatable, chronic medical disease involving complex interactions among brain circuits, genetics, the environment, and an individual’s life experiences. People with addiction use substances or engage in behaviors that become compulsive and often continue despite harmful consequences. Prevention efforts and treatment approaches for addiction are generally as successful as those for other chronic diseases.

**Diagnostic and Statistical Manual (DSM) 5-TR: Substance Use Disorder** (2022)

Source: <https://www.psychiatry.org/psychiatrists/practice/dsm>

Substance use disorders span a wide variety of problems arising from substance use, and cover 11 different criteria:

1. Taking the substance in larger amounts or for longer than you're meant to
2. Wanting to cut down or stop using the substance but not managing to
3. Spending a lot of time getting, using, or recovering from use of the substance
4. Cravings and urges to use the substance
5. Not managing to do what you should at work, home, or school because of substance use
6. Continuing to use, even when it causes problems in relationships
7. Giving up important social, occupational, or recreational activities because of substance use
8. Using substances again and again, even when it puts you in danger
9. Continuing to use, even when you know you have a physical or psychological problem that could have been caused or made worse by the substance
10. Needing more of the substance to get the effect you want (tolerance)
11. Development of withdrawal symptoms, which can be relieved by taking more of the substance

The DSM-5-TR allows clinicians to specify how severe or how much of a problem the substance use disorder is, depending on how many symptoms are identified.

- **Mild**: Two or three symptoms indicate a mild substance use disorder.^5^
- **Moderate**: Four or five symptoms indicate a moderate substance use disorder.
- **Severe**: Six or more symptoms indicate a severe substance use disorder.

**WHO International Classification of Disease (ICD) Disorders due to substance use**

ICD-10 <https://icd.who.int/browse10/2019/en#/F10-F19>

ICD-11 <https://icd.who.int/browse/2024-01/mms/en#1602669465>

**NOVA 4 Definition of Ultra Processed Foods (UPF)**

Ultra-processed foods are industrial formulations made entirely or mostly from substances extracted from foods (oils, fats, sugar, starch, and proteins), derived from food constituents (hydrogenated fats and modified starch), or synthesized in laboratories from food substrates or other organic sources (flavor enhancers, colors, and several food additives used to make the product hyper-palatable). Manufacturing techniques include extrusion, moulding and preprocessing by frying. Beverages may be ultra-processed.

(Source: <https://ecuphysicians.ecu.edu/wp-content/pv-uploads/sites/78/2021/07/NOVA-Classification-Reference-Sheet.pdf>)
